# Diagnostic accuracy of sFlt1/PlGF ratio as a marker for preeclampsia

**DOI:** 10.1186/s12884-020-2744-2

**Published:** 2020-02-07

**Authors:** Pooneh Nikuei, Minoo Rajaei, Nasibeh Roozbeh, Fatemeh Mohseni, Fatemeh Poordarvishi, Mohsen Azad, Solmaz Haidari

**Affiliations:** 1 Fertility and Infertility Research Center, Hormozgan University of Medical Sciences, Bandar Abbas, Iran; 2 Mother and Child Welfare Research Center, Hormozgan University of Medical Sciences, Bandar Abbas, Iran; 3 Molecular Medicine Research Center, Hormozgan Health Institute, Hormozgan University of Medical Sciences, Bandar Abbas, Iran; 4 Department of Biostatics, Faculity of ParaMedicine, Hormozgan University of Medical Sciences, Bandar Abbas, Iran

## Abstract

**Background:**

Preeclampsia (PE) is a serious complication of pregnancy and one of the main causes of maternal and neonatal mortality and morbidity in the world. Finding a biomarker with high sensitivity and specificity could lead to prediction and early diagnosis of the disease and reduces its complications. In this study, we evaluated diagnostic accuracy of Soluble fms-like tyrosine kinase-1 (sFlt-1) to Placental growth factor (PlGF) ratio for diagnosis of PE.

**Methods:**

The cases included 23 mild, 15 severe preeclamptic patients, and 20 normal term pregnant women as control referred to GYN ward of the Persian Gulf Hospital in Bandar Abbas from 2014 to 2016. Levels of sFlt-1 and PlGF were measured. Receiver Operating Characteristic (ROC) curve analysis was applied to calculate diagnostic accuracy of sFlt-1/PlGF ratio.

**Results:**

The mean Level of sFlt-1/PlGF in PE patients (91.33 ng/ml) was significantly higher than control women (17.62) (*P*<0.001). ROC curve analysis showed sFlt-1/PlGF ratio diagnostic accuracy in preeclamptic patients with Area Under Curve (AUC) of 0.90, the best cut-off value of 24.96, sensitivity and specificity of 84.2 and 85.0%, respectively.

**Conclusions:**

Our data showed sFlt-1/PlGF ratio has higher accuracy for differentiating PE patients from non-PEs in comparison with its power for differentiating severe or early onset forms of the disease.

## Background

Preeclampsia (PE) is diagnosed after 20 weeks of gestation [1]. This multisystem disorder affects 2–7% of pregnant women [2]. PE has serious complications including liver and kidney dysfunction, seizure and even death. PE is one of the major causes of maternal death, neonatal mortality and premature deliveries [3, 4]. As long as the placenta is present, PE is likely to happen and the recovery process starts post-delivery following the removal of placenta [5]. PE is divided into early-onset and late-onset according to the time of onset. Early-onset PE develops before 34 weeks of gestation, while late-onset PE develops at or after 34 weeks of gestation. The etiology of early-onset form of PE is related to incomplete trophoblast invasion and failure of normal spiral artery remodeling. Late-onset PE is associated with increased maternal vascular susceptibility to the normal inflammatory state of pregnancy or atherosis of a placenta grown initially normal [6]. Diagnostic criteria for PE include hypertension after 20 weeks of gestation and the coexistence of one or more of the following new-onset conditions: proteinuria, maternal organ dysfunction, and uteroplacental dysfunction [7]. Definite pathogenesis of PE is not completely clear, but the imbalance between angiogenic factors like vascular endothelial growth factor or placental growth factor (PlGF) and anti-angiogenic factors like soluble fms-like tyrosine kinase 1 (sFlt-1) are known to be related to the disease pathogenesis [8, 9].

In normal pregnancies, the level of sFLT-1 starts to rise after 30–32 weeks of gestation and PlGF level starts to decrease after 30 weeks of gestation. Actually cellular stress in the syncytiotrophoblast, which occurs during the last 8–10 weeks of a pregnancy leads to biochemical changes in levels of sFlt-1 and PlGF in normal pregnancies [10]. Circulating levels of sFlt-1 and PlGF alter in PE patients. This alteration begins before the disease onset and stays during the course of the disease [11]. In women with PE sFlt-1 rises approximately 5 weeks prior to disease onset [12] while the level of PlGF decreases before the rising of sFlt-1 [12, 13]. Hence, to improve the quality of PE diagnosis, some studies suggest sFlt1/PlGF Ratio as a better marker compared to measuring sFlt1 or PlGF separately for diagnosis of PE [11]. The aim of this study was to evaluate the diagnostic accuracy of sFlt-1: PlGF ratio, and the simplified best cut-off values for sFlt-1:PlGF ratio in assessing the diagnosis of PE.

## Methods

This research was a case-control study. Subjects were chosen from pregnant women referred to the Persian Gulf hospital located in Bandar Abbas in the south of Iran from 2014 to 2016. There were 38 PE patients and 20 normal term pregnant women as controls in the study. Informed written consent was obtained from all participants according to the protocols approved by the Ethical Committee of Hormozgan University of Medical Sciences (No. 1-HEC-93-7-8). Patients were sub-grouped as mild (23 women) and sever (15 women). Patients were classified into early-onset (9 women) and late-onset (29 women). Patients with diabetes, renal disease, collagen vascular diseases, and chronic hypertension were excluded from the study. PE was defined as gestational hypertension (systolic pressure > 140 mmHg or diastolic blood pressure > 90 mmHg on two or more occasions after gestational week 20) with proteinuria (> 0.3 g/day). The disease was defined severe if, more than one of the following criteria were met: (i) severe gestational hypertension; that is, systolic pressure > 160 mmHg or diastolic blood pressure > 110 mmHg on two or more occasions after gestational week 20, (ii) severe proteinuria; that is, protein ≥5 g in a 24-h urine specimen, (iii) oliguria, (iv) cerebral or visual disturbances, (v) pulmonary edema or cyanosis, (vi) epigastric or right upper quadrant pain, (vii) impaired liver function, (viii) thrombocytopenia or (ix) fetal growth restriction [1]. PE was defined early-onset (< 34 weeks of gestation) and late-onset (≥34 weeks) [14]. The whole blood samples were obtained from participants and centrifuged at 3000 g for 20 min and separated serum samples stored at − 80 °C for ELISA. Levels ofsFlt-1 and PlGF were measured using (ELISA, R&D System, Human VEGF R1/Flt-1Immunoassay, Catalog Number DVR100B and Human PlGF Immunoassay, Catalog NumberDPG00) according to the procedure provided by the manufacturer.

### Statistical analysis

One-way ANOVA/Kruskal-Wallis test with Bonferroni correction was applied for comparison of data between multiple groups and Mann-Whitney test / unpaired t-test was used for comparison between two groups based on the normality of data. Data were shown as number (%), mean (±SD). The Receiver Operating Characteristics (ROC) curve analysis was applied to calculate the Area Under the Curve (AUC) and to find the best cut-off point, Positive Predictive Values (PPVs), Negative Predictive Values (NPVs), diagnostic accuracy, sensitivity, specificity and likelihood ratios.

## Results

We did not observe any significant difference in maternal age (*P* = 0.546) and BMI before pregnancy (*P* = 0.355) between case and control groups. This showed that case and control groups were matched. Clinical characteristics are displayed in Table 1. The number of nulliparous women in both groups was almost similar (40% in the control group and 44.7% in the case group). Recurrent PE was observed in 15.8% of women in the case group.

**Table 1:** Clinical characteristics of the participants

Characteristics	PE (38)	Controls (20)	*P*-value
Maternal age (year)	27.18 ± 5.86	26.25 ± 4.93	0.546
Placental weight(g)	423.95 ± 87.37	613.50 ± 53.44	< 0.001*
BMI before pregnancy (kg/m2)	23.81 ± 3.98	23.21 ± 3.50	0.355
Systolic Blood Pressure (mmHg)	150.68 ± 1.61	110.23 ± 0.70	< 0.001*
Diastolic Blood Pressure (mmHg)	100.07 ± 0.95	60.70 ± 0.66	< 0.001*

Results are presented as mean ± SD. *P*<0.05 is considered significant

**Fig. 1:**
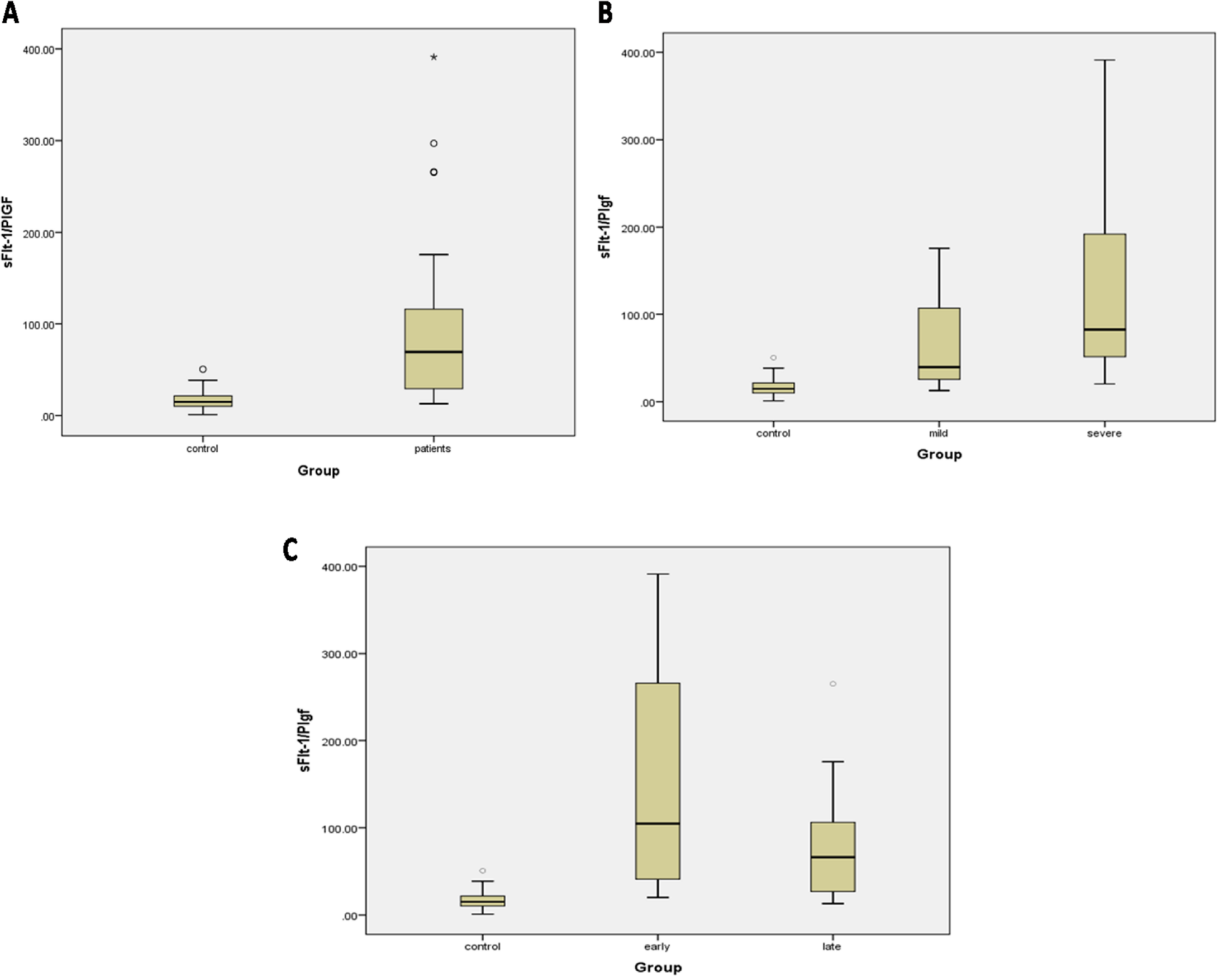
**a** Comparison of sFlt-1/PlGFratio between PE patients and controls; **b** mild and sever PE patients with controls; **c** early and late onset PE patients with controls, which shows significant increase in all PE groups in comparison with the normal women. The 10th and 90th centiles are represented by lower and upper bars. The horizontal line in the box shows median

Mean Level of sFlt-1/PlGF was 91.33 ng/ml in PE patients and 17.62 in controls which were increased significantly (*P*<0.001). In comparison between mild, severe and controls the mean level of sFlt-1/PlGF was 130.97 in severe PE patients which was significantly increased compared to controls (*P*<0.001). Also, it was 65.48 in mild PE patients which was increased significantly compared to controls (*P*<0.001). Although the level of sFlt-1/PlGF was higher in severe PE patients compared to mild women, there was no significant difference between these two groups (*P* = 0.389). Also in the comparison between early-onset, late-onset and controls the mean level of sFlt-1/PlGF was 153.44 in the early-onset PE patients and 72.06 in late-onset PE patients. There was a significant difference between early-onset group and controls (*P*<0.001), and also between late-onset PE patients and controls (*P*<0.001), but there was no significant difference between early and late onset PE patients (0.503) (Fig. 1).

### ROC-curve analysis

**Fig. 2:**
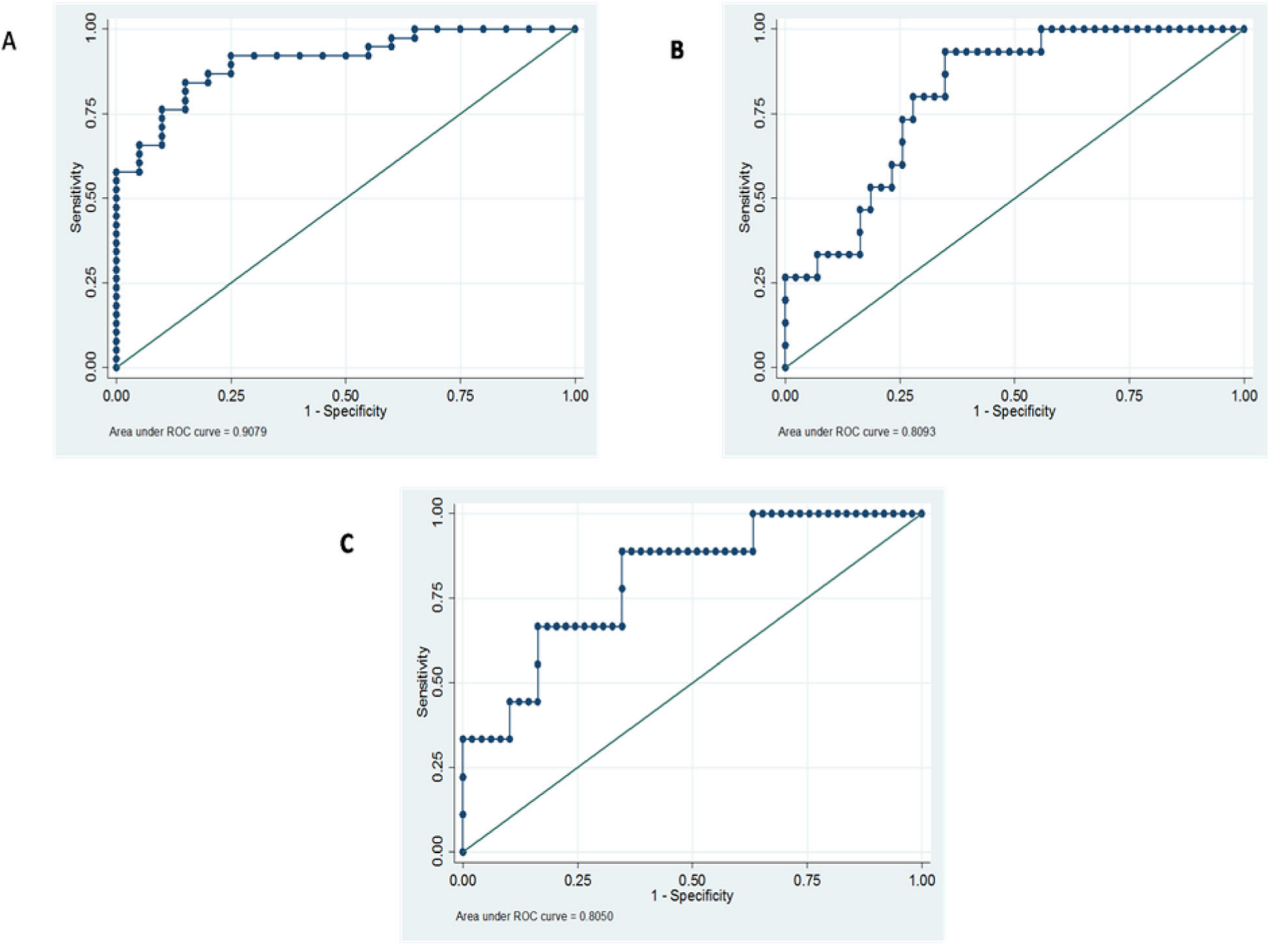
ROC curve analysis of sFlt-1/PlGF ratio in preeclampsia, which shows the ability of maternal serum sFlt-1/PlGF ratio to (**a**) differentiate preeclampsia from normal pregnancies; (**b**) severe PE patients; (**c**) early-onset PE patients. sFlt-1 has the highest AUC for the diagnosis of PE patients from normal pregnant women

The ROC curve analysis was applied to differentiate PE patients from normal controls and also for differentiating severe and early-onset forms of PE using sFlt-1/PlGF ratio. The results for differentiation of PE patients from normal pregnancies showed an AUC of 0.90 (95% CI =0.83–0.98). The best cut-off value was 24.96 ng/ml with sensitivity of 84.2% (95% CI =68.7–94) and specificity 85% (95% CI =62.1–96.8). Table 2 and Fig. 2 provide results related to LR, PPV, and NPV. Moreover, ROC curve analysis was also applied to differentiate early onset PE from controls using sFlt-1/PlGF ratio (Table 2 and Fig. 2).

**Table 2:** Diagnostic accuracy of sFlt/PlGF ratio for diagnosing preeclampsia

Groups	cut off	Sensitivity	Specificity	LR+	LR-	PPV	NPV	Area (95% CI)
PE	24.96	84.2(68.7–94)	85(62.1–96.8)	5.61	0.186	91.4	73.9	90(0.83–0.98)
Sever PE	40.9	80(51.9–95.7)	72.09(56.3–84.7)	2.8	0.27	50	91.2	0.80(0.65–0.95)
Early-onset PE	39.7	88.89(51.8–99.7)	65.31(50.4–78.3)	2.56	0.17	32	97	0.80(0.65–0.95)

*LR* Likelihood Ratio, *PPV* Positive Predictive Value, *NPV* Negative Predictive Value, *CI* Confidence Interval

## Discussion

Anti-angiogenic factors like sFlt-1 are released by the placenta in the circulation of women affected to PE and are known as the cause of endothelial dysfunction observed in this disease. This anti-angiogenic protein inhibits the activity of VEGF and PlGF and results in hypertension and proteinuria as main symptoms of PE [11]. Recombinant sFlt-1 could block development of endothelial tubes and inhibit vasodilatory effects of VEGF and PlGF in vasculature [15]. In women with PE, the levels of sFlt-1 rise 5 weeks prior to clinical presentations, and stay elevated till the onset of the disease [12, 16] while the serum PlGF decreases before clinical presentation [12]. So these two markers could be used for prediction and diagnosis of PE, but recent studies showed that sFlt1/PlGF ratio is a better marker with more accuracy for screening PE rather than sFlt-1 or PlGF alone [17].

According to our knowledge, this is the first study which investigates the diagnostic accuracy of sFlt1/PlGF ratio in Iranian women with PE. Results of our study showed sFlt1/PlGF ratio has the highest accuracy for differentiating PE patients from normal pregnant women. In our study the best cut-off for the highest sensitivity (84.2%) and specificity (85%) was 24.96 ng/ml with LR^+^ 5.61, LR^−^ 0.186 for differentiating PE from healthy women. So because of high sensitivity, specificity, positive and negative LR of sFlt1/PlGF ratio, it could help in diagnosing high-risk women for developing PE and decreasing costs related to serious maternal and neonatal complications by precise observation and early medical interventions. Also, we evaluated the accuracy of sFlt1/PlGF ratio in diagnosing severe and early-onset PE which more likely lead to preterm delivery.

Based on our ROC curve analysis for sFlt1/PlGF ratio, the best cut-off for diagnosing severe PE patients is 40.9 ng/ml with 80% sensitivity and 72.09% specificity, and the best cut-off for diagnosing early-onset PE is 39.7 ng/ml with sensitivity of 88.89% and specificity of 65.31%. In this study, sFlt1/PlGF ratio showed the highest sensitivity and specificity in differentiating PE patients from normal pregnant women. Our results are in agreement with findings of other studies which reported association of sFlt1/PlGF ratio with clinical diagnosis of PE [4, 8, 12, 18, 19].

Lim et al. reported higher levels of sFlt-1/PlGF in women with preeclampsia compared to normal controls. Like our study, they reported sFlt-1/PlGF ratio was not significantly different between mild and severe PE patients. Also, the cut-off value of sFlt-1/PlGF was 20.5 with AUC of 0.85 for the prediction of preeclampsia [8]. DE VIVO et al. applied ROC curve analysis for the second-trimester marker values in PE patients. They showed the best diagnostic profile for sFlt-1/PlGF ratio with AUC of 0.92 and the best cut-off of 38.47. They found this marker with the best prediction power for pre-eclampsia with a specificity, a sensitivity, a diagnostic accuracy, a PPV and a NPV of 88.5%, a positive likelihood ratio of 7.7 and a negative likelihood ratio of 0.13 [20]. KUSANOVIC et al. reported the best predictive performance for sFlt1/PlGF ratio with a sensitivity of 100%, specificity of 98–99%, and likelihood ratios for a positive test of 57.6, 55.6 and 89.6, respectively, for predicting early-onset preeclampsia [21].

Rana *et. al*. reported sFlt1/PlGF ratio of more than 85 had an association with harmful pregnancy complications and termination of pregnancy in 2 weeks. Moreover, they reported sFlt1/PlGF ratio along with systolic blood pressure (SBP) and proteinuria were a better predictive tool rather than SBP, proteinuria, and uric acid levels [3]. Ohkuchi et al. showed the best diagnostic power of sFlt1/PlGF ratio for both early and late onset PE. They reported a cut-off value of 45 with 97 and 95% sensitivity and specificity respectively for diagnosis of all preeclampsia and for diagnosis of early-onset PE (100 and 95%) [22].

Meanwhile, sFlt1/PlGF ratio could be a useful marker for the differential diagnosis of PE [3]. Hypertension and proteinuria are hallmark symptoms of both preeclampsia and chronic kidney disease (CKD). It is important to differentiate PE from CKD and manage PE efficiently. Rolfo et al. found that serum levels of sFlt1/PlGF ratio significantly increase in PE compared with CKD and controls [23]. Our results support previous studies about the association of high sFlt-1/PlGF ratio with the diagnosis of PE and also severe and early-onset forms of the disease. This marker has the highest sensitivity and specificity for distinguishing PE from normal pregnancies in comparison to severe and early-onset forms of the disease. Using angiogenic biomarkers might lead to screening and faster diagnosis of PE patients and also to preventing them from serious complications. One of the limitations of this study was the sample size. More studies in larger populations and among other ethnicities are required to determine the cost-effectiveness of sFlt-1/PlGF ratio as a biomarker for PE diagnosis. Another limitation was that it was not possible to carry out sampling prior to the patients’ diagnosis and samples were taken after PE diagnosis.

## Conclusions

Our data showed that sFlt-1/PlGF ratio has the highest accuracy for the diagnosis of PE patients from normal pregnant women in comparison to its power for the diagnosis of severe or early-onset forms of the disease.

## Data Availability

The datasets used and/or analyzed during the current study are available from the corresponding author on reasonable request.

## Abbreviations

PE: Preeclampsia
PlGF: Placental growth factor
ROC curve: Receiver operating characteristic curve
sFlt-1: Soluble fms-like tyrosine kinase-1
